# Posttraumatic stress disorder and total amyloid burden and amyloid-β 42/40 ratios in plasma: Results from a pilot study of World Trade Center responders

**DOI:** 10.1016/j.dadm.2019.01.003

**Published:** 2019-02-28

**Authors:** Sean A.P. Clouston, Yael Deri, Erica Diminich, Richard Kew, Roman Kotov, Candace Stewart, Xiaohua Yang, Sam Gandy, Mary Sano, Evelyn J. Bromet, Benjamin J. Luft

**Affiliations:** a Department of Family, Population, and Preventive Medicine, Program in Public Health, Stony Brook University, Stony Brook, NY; b Department of Medicine, Stony Brook University, Stony Brook, NY; c Program in Public Health, Stony Brook University, Stony Brook, NY; d Department of Pathology, Stony Brook University, Stony Brook, NY; e Department of Psychiatry, Stony Brook University, Stony Brook, NY; f Departments of Neurology and Psychiatry, Icahn School of Medicine at Mount Sinai, New York NY; g Department of Psychiatry, Icahn School of Medicine at Mount Sinai, New York City, NY; h James J Peters VAMC, Bronx NY

**Keywords:** World Trade Center disaster, Posttraumatic stress, Cognitive impairment, Plasma markers of neuropathology, Amyloid burden, Tau, Neurofilament-light

## Abstract

**Introduction:**

Chronic posttraumatic stress disorder (PTSD) is associated with poor memory and increased burden of various degenerative cerebral neuropathologies. The goal of this pilot study was to determine whether PTSD was associated with changes in plasma-based neuropathological biomarkers of neurodegeneration among World Trade Center (WTC) responders.

**Methods:**

Thirty-four WTC responders had blood drawn and flash-frozen within 15 minutes of retrieval. PTSD symptoms were assessed at that time. Age, sex, and WTC exposure duration were obtained from medical records. Plasma was assayed in duplicate using an ultra-sensitive single-molecule enzyme-linked immunosorbent assay to examine the distribution of amyloid-β (Aβ) 42/40 ratios, total Aβ, total tau, and neurofilament light (NfL). The comparison group was drawn from a bank of healthy controls collected and assayed at the same facility.

**Results:**

The average age of WTC responders at blood draw was 53 years. Half were PTSD positive (PTSD+) as indicated by symptom severity. WTC responders had lower Aβ42/Aβ40 ratios but higher total tau and NfL levels in the plasma than healthy controls. PTSD+ status was associated with lower plasma Aβ load and higher Aβ42/Aβ40 ratios.

**Discussion:**

Findings suggest that PTSD may be associated with alterations in plasma markers related to Aβ, tau, and NfL, highlighting the potential association between PTSD status and neurodegenerative neuropathology in WTC responders.

Posttraumatic stress disorder (PTSD) is characterized by reexperiencing stressful memories, while sleeping or awake, that elicit a physiological stress response and cause emotional distress [1]. The prevalence of PTSD varies, but after 9/11/2001, the prevalence among World Trade Center (WTC) responders, the focus of this study, was 17.6% [2]. Recent animal studies revealed that exposure to severe and chronic stress can elicit amyloid-β (Aβ) deposition in amyloid precursor protein transgenic mice [3]. In other studies, inhalation of metal vapor was shown to cause a rapid doubling of endogenous brain Aβ42 in mice [4]. In humans, *in vivo* neuroimaging studies have also revealed that chronic PTSD is associated with hippocampal volume loss and cortical thinning [5], [6]. Although these findings are suggestive of the presence of a degenerative neuropathological process causing brain changes, the only previous study that focused on discovery of possible associations between PTSD and neuropathology used positron emission tomography to show that PTSD symptom severity was correlated with increased retention of an amyloid-binding ligand, presumably indicating increased Aβ load [7]. Positron emission tomography studies are invasive and costly. The goal of the present pilot study was to evaluate these associations by examining associations between PTSD symptom severity and plasma-based markers of Aβ burden, total tau, and neurofilament light (NfL) in a sample of WTC responders.

## 1 Methods

### 1.1 Participants

The sample included 34 WTC responders who enrolled in the Stony Brook University WTC Health and Wellness study, a prospective study of men and women who responded to the WTC events on and after 9/11/2001 who are monitored as part of an ongoing program funded by the Centers for Disease Control and Prevention [8]. Individuals with WTC or military-related head injuries were excluded from this study. Blood was banked as part of ongoing monitoring efforts and retrieved here to facilitate analysis of biomarkers. Plasma for this study was retrieved in a subsample for this analysis because these responders had clinical histories with complete information on a range of variables of interest including probable PTSD status, exposure severity, and demographics. A balanced sample of 50% with and without PTSD was identified. Plasma samples were available for all 34 subjects. However, there were missing values for tau (*n* = 1), Aβ (*n* = 1), and NfL (*n* = 3) due to contaminants in the plasma at the time of assay.

### 1.2 Ethics

The study received approval from the Stony Brook Institutional Review Board (IRB, CORIHS #604113). All participants provided written informed consent.

### 1.3 PTSD measure

PTSD symptom severity was measured using the 17-item PTSD checklist (PCL-17) modified to fit experiences specific to the WTC events [9]. For this measure, respondents were asked to rate the degree to which they were bothered by each PTSD symptom in the past month, with answers ranging from not at all (1) to extremely (5). Internal reliability was excellent (α = 0.87). To identify probable PTSD (PTSD+), a cutoff of 40 was applied [10].

### 1.4 Covariates

Age at 9/11/2001 and at time of blood draw and sex of responders were recorded in the medical record. Exposure was assessed during the first monitoring visit. Exposure chronicity was based on duration of work in the WTC area during the months after 9/11 and was dichotomized into high (>15 weeks exposure) versus low (0-15 weeks).

### 1.5 Collection methods

Whole blood samples from WTC responders were collected in K2-EDTA blood collection tubes (BD Vacutainer, Franklin Lakes, NJ) and placed on ice and then centrifuged at 2000g for 15 minutes at 4°C. Plasma samples were separated and placed into polyethylene tubes before being stored at −80°C until shipping. Plasma samples to be assayed at Quanterix laboratories were transported in dry ice package.

### 1.6 Assay technologies

Concentrations of three biomarkers were analyzed using Simoa, a high-definition analyzer that is a bead-based enzyme-linked immunosorbent assay (ELISA). The kit includes monoclonal anti-Aβ40, anti-Aβ42, anti–total tau, and anti-NfL antibodies that are directed at each of the target peptides. The Aβ40, Aβ42, total tau, and NfL assays have lower limits of detection of 0.196, 0.045, 0.019, and 0.104 pg/mL, respectively. Because the median healthy donor in the Quanterix databases had Aβ40, Aβ42, total tau, and NfL concentrations exceeding 100 times that level, levels of detection were well within the measurable limit. The average coefficients of variation (intraplate and interplate) values were below 10%. Samples were auto-diluted at 4X. Here, concentrations based on dilution using data from two control subjects were reported. Results reported represent averaged results across duplicate arrays. Among reported results, no individuals were deemed to have plasma biomarker levels below the lower limit of quantification. Normative data among healthy individuals (*N* = 20) were retrieved from a publicly available commercial biobank and separately assayed by Quanterix; median values were total 220.1 pg/mL, 5.3%, 1.4 pg/mL, and 5.3 pg/ml for Aβ load, Aβ42/Aβ40 ratios, total tau, and NfL, respectively.

## 2 Statistical analysis

Descriptive statistics for all demographic and clinical variables were calculated using Stata 15/SE (StataCorp). Comparisons were made between the two groups using χ^2^ statistics for categorical variables and Student's *t*-tests for continuous variables. Standardized mean differences (∂) were used as a measure of effect size. *P*-values were reported to determine statistical significance. Medians were used to compare WTC responders with published control data using the Mann-Whitney test; χ^2^ statistics and *P*-values were reported.

## 3 Results

The sample (Table 1) consisted of 34 WTC responders who were primarily male and were, on average, 53 years old at the time of the blood draw. In addition, the PTSD+ and PTSD− groups did not differ significantly in age, gender, or exposure duration (Table 1). Not shown in this table, the level of association across different neurological biomarkers was only significant when comparing total Aβ load with Aβ42/Aβ40 ratios (r = −0.59, *P* < .001). Assay values for WTC responders had lower Aβ42/Aβ40 ratios (χ^2^ = 11.3*, P* = .001) and higher total tau (χ^2^ = 16.7*, P* < .001) and NfL (χ^2^ = 6.4*, P* = .012) burden than those for controls.

**Table 1:** Characteristics of sampled WTC responders

Characteristics of interest	Overall sample (N = 34)	
	ES	SD
Characteristic		
Age at 9/11/2001, years	38.19	7.93
Age at blood draw, years	52.52	8.02
>15 weeks at WTC, %	20.59	
Female, %	6.25	
Biomarker		
Aβ42/Aβ40 ratio, %	4.59	1.36
Total Aβ, pg/mL	216.95	50.39
Total tau, pg/mL	2.67	0.91
NfL, pg/mL	8.97	3.65

Abbreviations: ES, estimate; SD, standard deviation; WTC, World Trade Center; Aβ, amyloid-β; NfL, neurofilament light; pg/mL, picograms per milliliter.

Group comparisons identified significant associations between PTSD and lower total Aβ levels (Table 2). Additionally, although not reaching statistical significance in group-wise analyses in this sample, increased PTSD symptom severity was associated with higher Aβ42/Aβ40 ratios (r = 0.36, *P* = .040).

**Table 2:** Differences between WTC responders with and without PTSD

Characteristics of interest	PTSD−		PTSD+		Difference	
	ES	SD	ES	SD	∂	*P*
Characteristic						
Age at 9/11/2001, years	37.75	9.03	38.63	6.94	−0.03	0.941
Age at blood draw, years	52.29	9.18	52.75	6.97	0.01	0.980
>15 weeks at WTC, %	17.65		23.53			0.536
Female, %	6.25		6.25			0.965
Biomarker						
Total Aβ, pg/mL	236.27	41.40	198.76	52.41	−0.79	0.031
Aβ42/Aβ40 ratio, %	4.17	0.27	4.98	1.82	0.62	0.089
Total tau, pg/mL	2.62	0.97	2.72	0.88	0.11	0.759
NfL, pg/mL	8.28	1.92	9.58	4.67	0.26	0.476

Abbreviations: ES, estimate; SD, standard deviation; ∂, standardized mean difference; WTC, World Trade Center; Aβ, amyloid-β; NfL, neurofilament light; pg/mL, picograms per milliliter.

## 4 Discussion

In this cross-sectional pilot study, we explored the potential for PTSD to associate with changes in plasma biomarkers indicative of neurodegenerative processes. We observed that PTSD may be associated with reduced plasma Aβ and increased Aβ42/Aβ40 ratios. Neurodegenerative processes can include a broad array of neuropathological changes as hallmarks of the disease [11]. For example, prior work in veterans has suggested that PTSD may increase risk of Alzheimer's disease (AD) [12] and dementia [13], whereas neuroimaging work has identified increased burden of brain Aβ among veterans with PTSD [7]. WTC responders with PTSD have also been shown to be at increased risk of AD-related deficits including poorer memory [14], cognitive impairment [15], and disturbances in neurologically associated movements [16].

The present study adds a novel investigation of plasma Aβ, total tau, and NfL concentrations as potential hallmarks of latent degenerative neuropathology using Simoa technology. We observed an overabundance of plasma tau in this cohort that was accompanied by high Aβ42/Aβ40 ratios when compared with healthy specimen. In addition, results suggest that reductions in total plasma Aβ load among those with PTSD+ may be accompanied by increasing Aβ42/Aβ40 ratios, indicating a larger than expected decrease in Aβ40 as a proportion of the decrease in total Aβ. Recent work using similar technologies to measure plasma Aβ found that decreased Aβ load was indicative of Aβ deposition in the brain [17]. This result may be because Aβ40, which is deposited in the brain from the blood, plays a critical role in modulating Aβ aggregation and dense-core plaque formation [18], potentially indicating a role in AD pathogenesis in this population [19]. Together, results suggest that PTSD may be associated with circulating biomarkers and perhaps parenchymal brain changes related or identical to those observed in AD.

### 4.1 Limitations

Although we sought to examine novel peripheral biomarkers of brain neuropathology, it is limited in a number of ways. Critically, it is a small cross-sectional pilot study of WTC-exposed individuals. We are reliant on peripheral fluid sampling to detect neuropathology using ultra-sensitive Simoa technology. Although measures have been validated in external samples, we have not yet validated correlations between peripheral neuropathological biomarkers and neuroimaging modalities (e.g., Aβ positron emission tomography) in this population. Despite having access to public control data, we lacked an external control group and cannot clarify the role of batch effects. We examined individuals with chronic PTSD more than a decade after the traumatic exposures. Insofar, as the latency period linking PTSD with neuropathology is long, this represents a strength. However, this delay also reflects a diminished extent to which results may be generalizable among recently traumatized individuals with acute PTSD. Although this study explicitly studies exposure to chronic PTSD, it is unclear to what extent the results of this study generalize to other stress-based experiences including exposures to chronic stressors at work or at home. We cannot exclude a possible role for inhaled toxins in this process [20]. Also, in the absence of quantitative postmortem neuropathology, we currently refrain from applying the diagnosis of AD in favor of more descriptive language (e.g., AD-like neuropathology, amyloidosis, and tauopathy).

## 5 Conclusions

This study is the first of its kind in a sample of WTC responders and is among the first to examine a multiplex array of plasma-based neurological biomarkers. Although notable results were found linking PTSD with plasma biomarkers that often reflect brain neuropathology, this study suggests that more research is warranted using neurological biomarkers longitudinally and on larger populations. Further work is warranted in this population to determine the extent to which plasma-based neuropathological biomarkers reflect brain parenchymal degenerative changes that underlie cognitive symptomatology present in WTC responders.

**Research in context**

1. Systematic review: A review of studies in PubMed using terms including posttraumatic stress disorder (PTSD) and cognitive, memory, amyloid-β (Aβ), or Alzheimer's disease (AD) revealed strong and consistent associations between PTSD and increased risk of symptoms of AD but identified little evidence about biomarkers of AD such as Aβ, protein-tau, or neurofilament light.
2. Interpretation: The findings of this study revealed that, among World Trade Center responders without concomitant head injury, increased PTSD was associated with decreased plasma Aβ load and increased Aβ42/Aβ40 ratios.
3. Future directions: These findings suggest that future evaluation of plasma-based neuropathological biomarkers is warranted and also support the use of positron emission tomography to determine whether altered plasma biomarkers of AD-related neuropathology occurring with chronic PTSD reflect an increased burden of Aβ in the cerebral cortex as is evident in AD.
